# Supplementary material for: Strongly ROS-Correlated, Time-Dependent, and Selective Antiproliferative Effects of Synthesized Nano Vesicles on BRAF Mutant Melanoma Cells and Their Hyaluronic Acid-Based Hydrogel Formulation
Source: Int J Mol Sci. 2024 Sep 19;25(18):10071. doi: 10.3390/ijms251810071 (PMC11432396; doi:10.3390/ijms251810071)
Supplement: Supplementary file 1 [file ijms-25-10071-s001.zip › ijms-3218102-supplementary.pdf]

International Journal of *Molecular Sciences*

MDPI

Supplementary Materials

Strongly ROS-Correlated, Time-Dependent and Selective Anti-proliferative Effects of Synthesized Nano Vesicles on BRAF Mutant Melanoma Cells and Their Hyaluronic Acid-Based Hydrogel Formulation

Silvana Alfei [1,*], Guendalina Zuccari [1,2], Constantinos M. Athanassopoulos [3], Cinzia Domenicotti [4,5], and Barbara Marengo [4,5,*]

[1]Department of Pharmacy, University of Genoa, Viale Cembrano, 16148 Genoa, Italy; guendalina.zuccari@unige.it (G.Z.)

[2]Laboratory of Experimental Therapies in Oncology, IRCCS Istituto Giannina Gaslini, Via G. Gaslini 5, 16147, Genoa, Italy

[3]Depatment of Chemistry, University of Patras, University Campus Rio Achaias, 26504 Greece; kath@chemistry.upatras.gr (C.M.A.)

[4]Department of Experimental Medicine (DIMES), University of Genova, Via Alberti L.B., 16132 Genoa, Italy; cinzia.domenicotti@unige.it (C.D.)

[5]IRCCS Ospedale Policlinico San Martino, Genova, Italy

* Correspondence: alfei@difar.unige.it (S.A.); Tel.: +39 010 355 2296; barbara.marengo@unige.it (B.M.)

Section S1. Biological results.

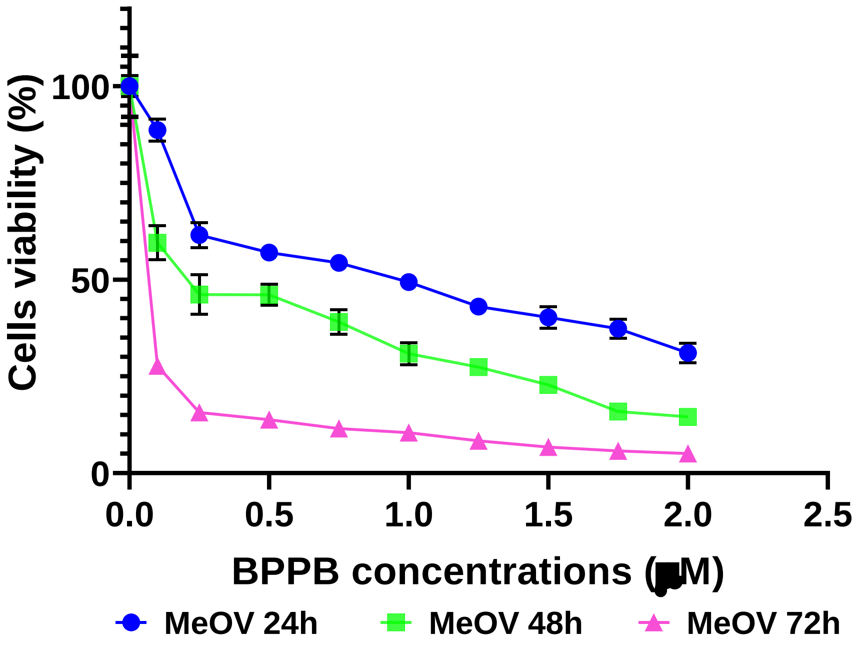

Figure S1. Cell viability (%) of MeOV vs increasing BPPB concentrations (0.1-2.0 μM) after 24 hours (blue line), 48 hours (green line) and 72 hours (pink line) of exposure. Concentration = 0.0 μM corresponded to the control.

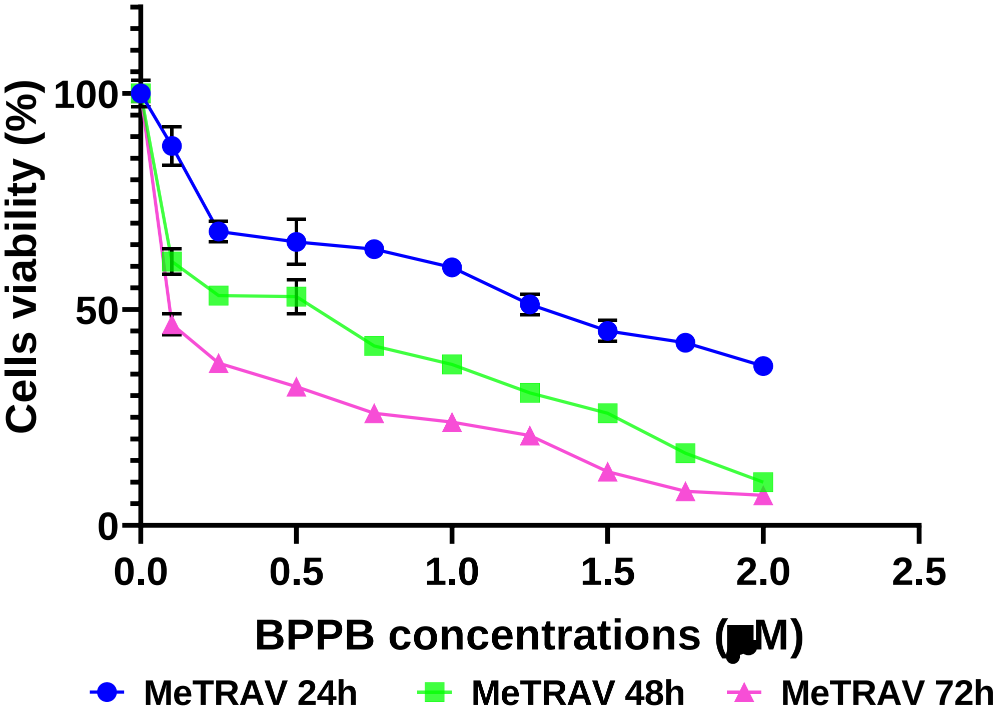

Figure S2. Cell viability (%) of MeTRAV vs increasing BPPB concentrations (0.1-2.0 μM) after 24 hours (blue line), 48 hours (greenline) and 72 hours (pink line) of exposure. Concentration = 0.0 μM corresponded to the control.

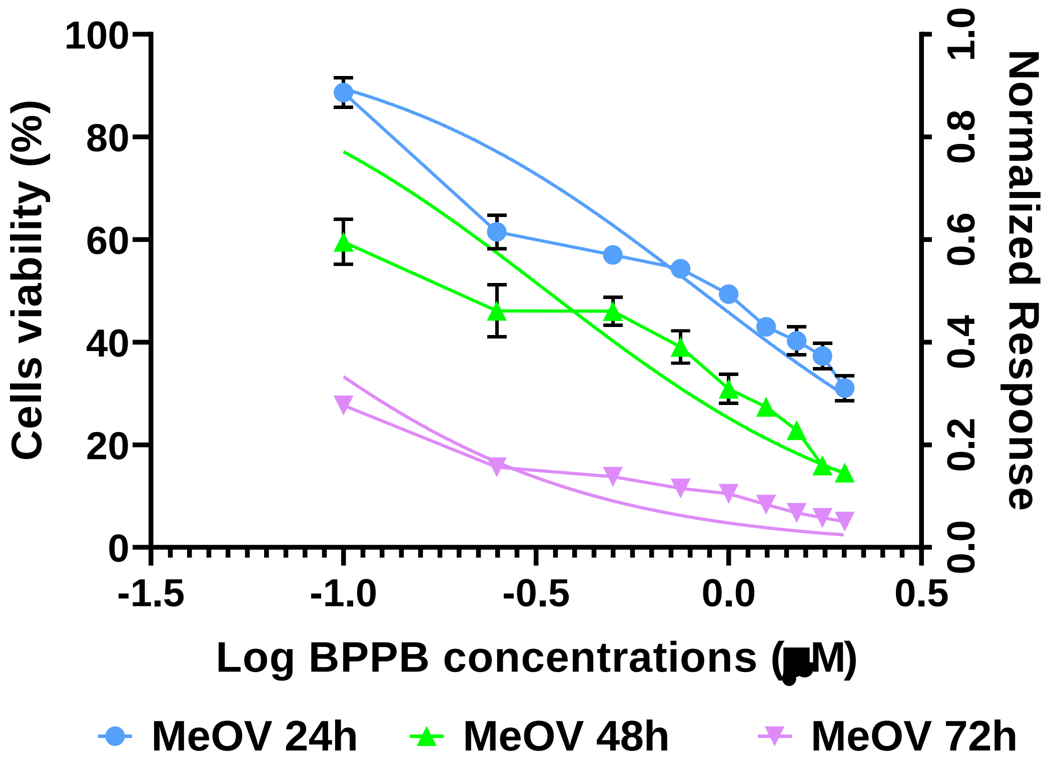

Figure S3. Plot of Log concentration of BPPB vs. cell viability (%) of MeOV after 24, 48, and 72 hours of exposure (blue, green and light purple traces with indicators and error bars) and plot of nonlinear fit of Log concentrations of BPPB vs. normalized response after 24, 48, and 72 hours of exposure (blue, green and light purple traces without indicators).

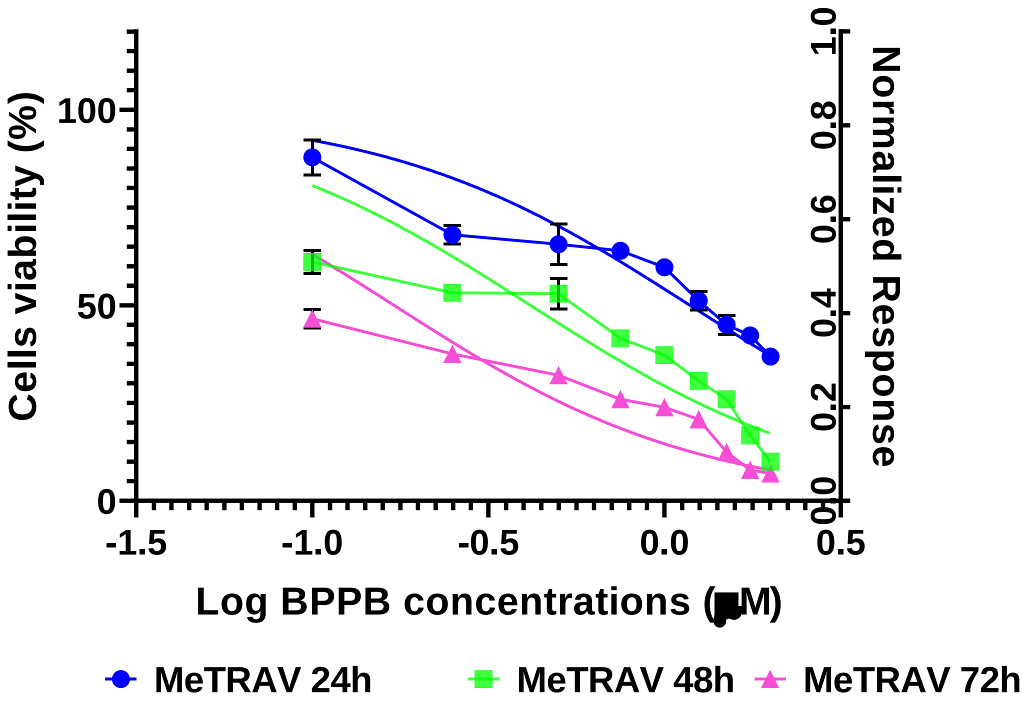

Figure S4. Plot of Log concentration of BPPB vs. cell viability (%) of MeTRAV after 24, 48, and 72 hours of exposure (blue, green and pink traces with indicators and error bars) and plot of nonlinear fit of Log concentrations of BPPB vs. normalized response after 24, 48, and 72 hours of exposure (blue, green and pink traces without indicators).

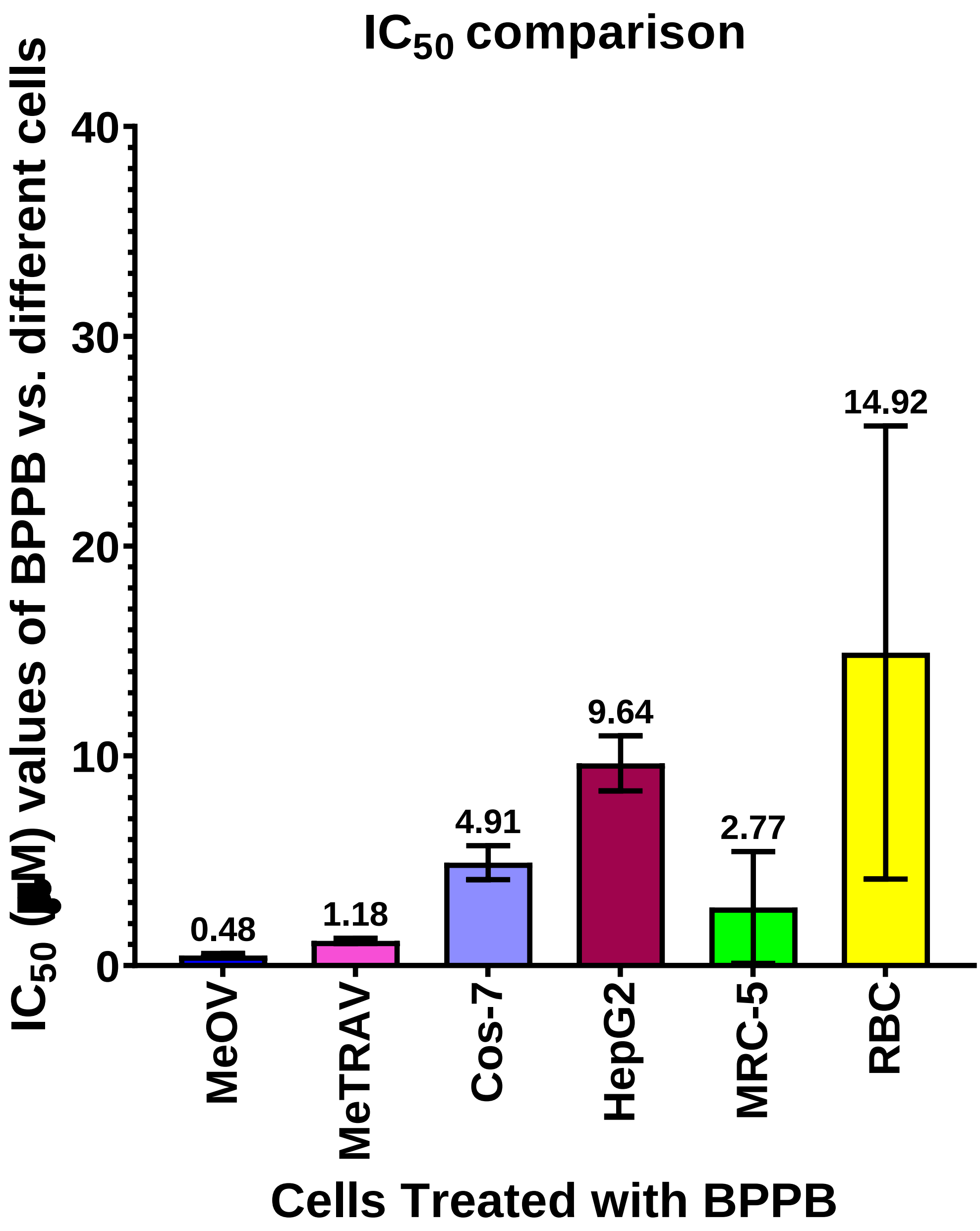

Figure S5. Comparison between the IC_{50} values determined on MM cells and those determined on three mammalian cell lines and RBCs after 24-huors treatment.

Section S2. Hydrogel Characterization.

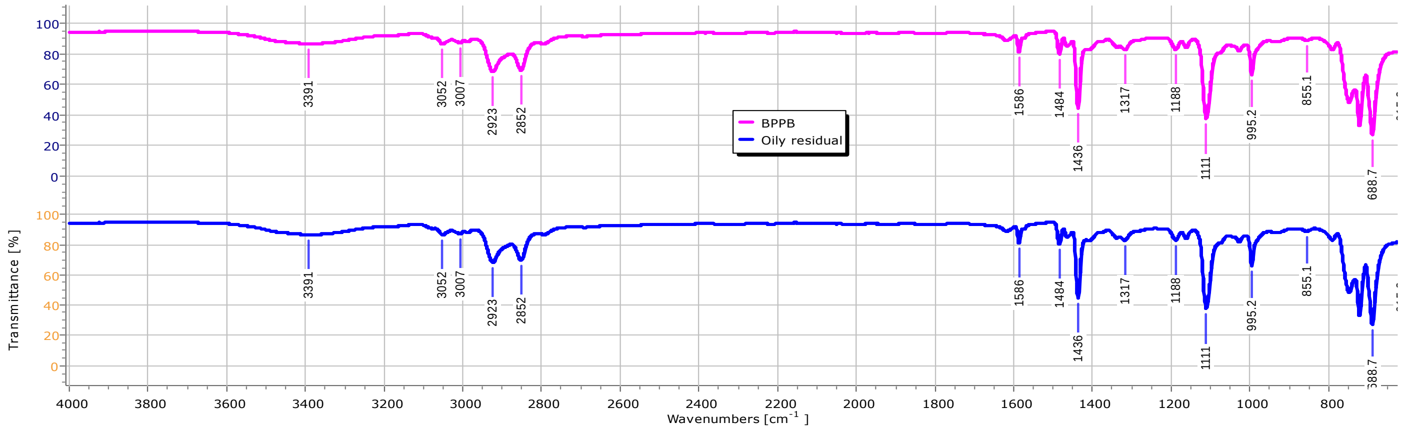

Figure S6. Comparison between the ATR-FTIR spectrum of pure BPPB and that of the oily residual recovered by the not absorbed water afforded during the preparation of HA-BPPB-HG.

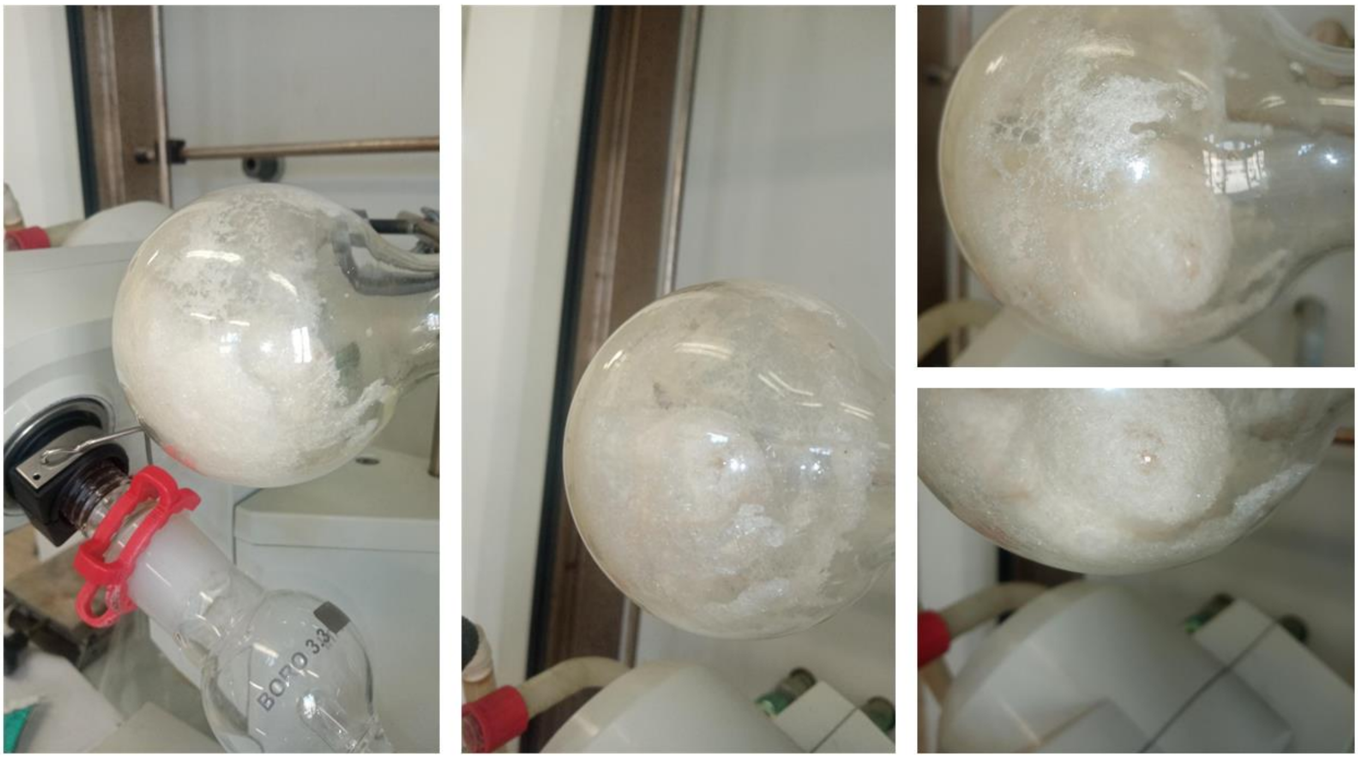

Figure S7. Appearance of the lyophilized HA-BPBB-HG (glassy state).

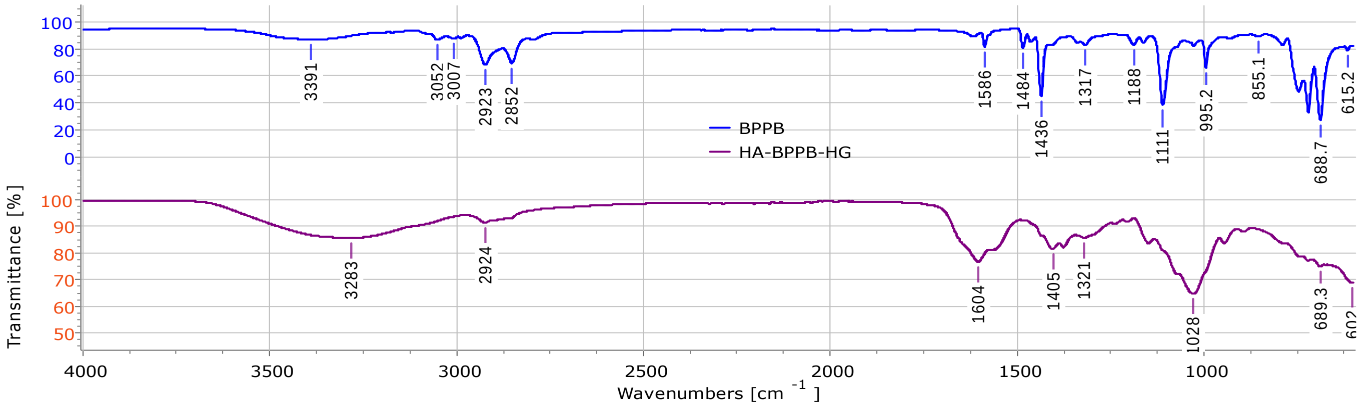

Figure S8. ATR-FTIR spectra of BPPB (blue line), HA and HA-BPPB-HG (dark purple line).

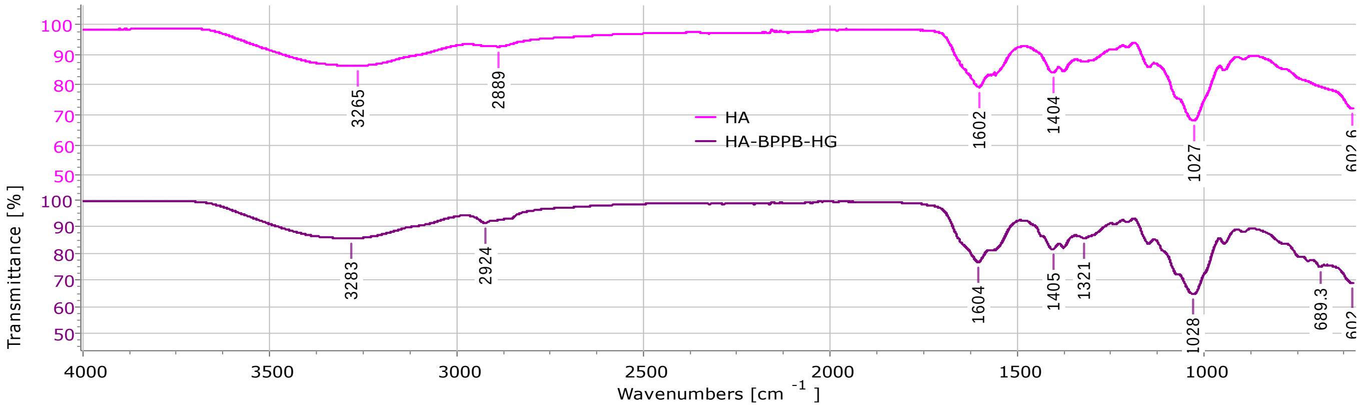

Figure S9. ATR-FTIR spectra of HA (fuchsia line) and HA-BPPB-HG (dark purple line).

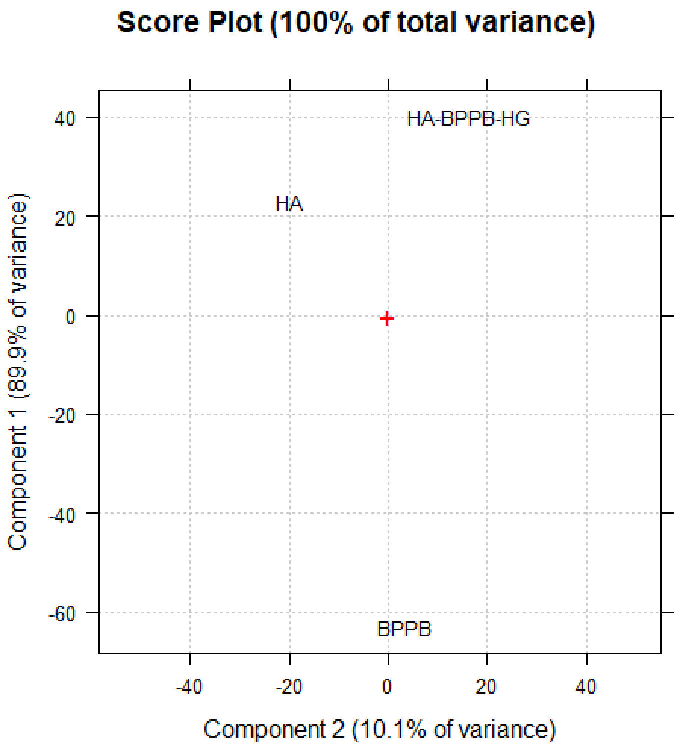

Figure S10. PCA results reported as PC2 vs PC1.

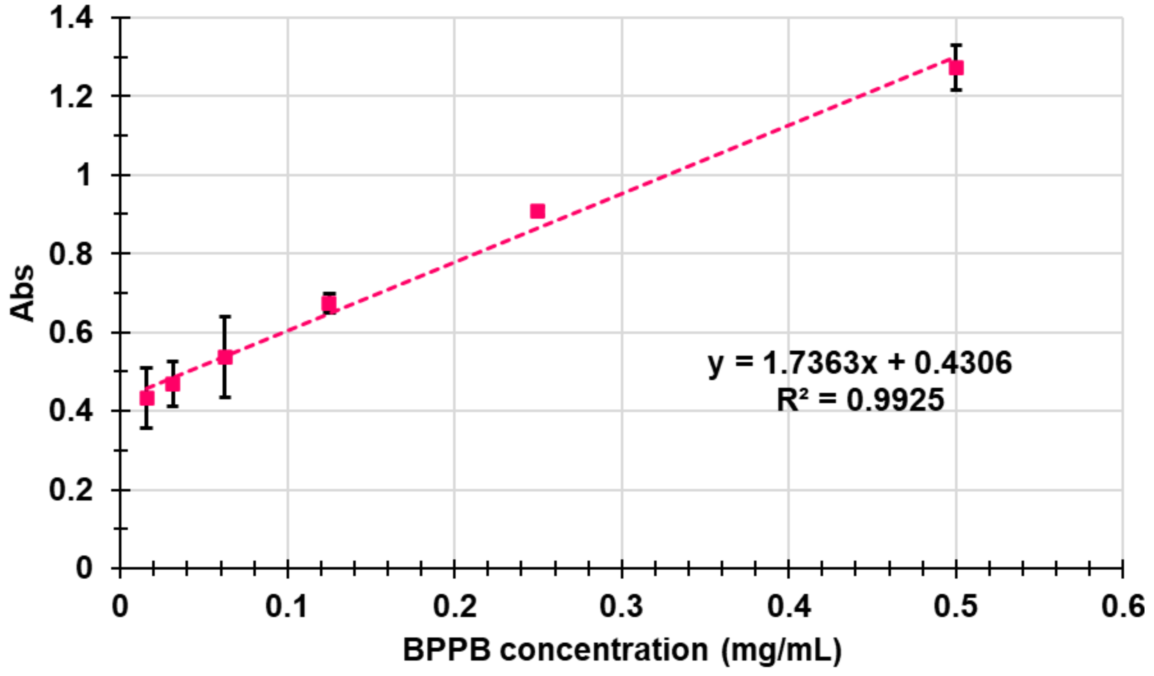

Figure S11. BPPB calibration curve.

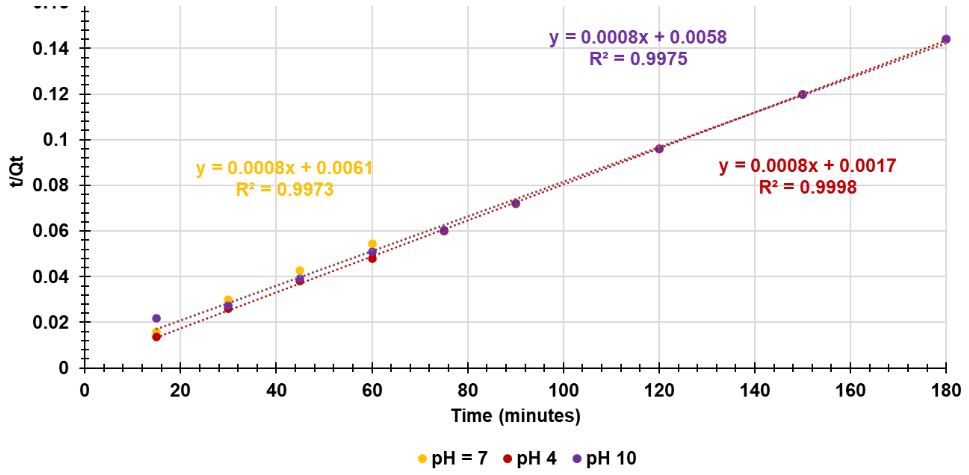

Figure S12. PSO kinetic model on swelling experiments.

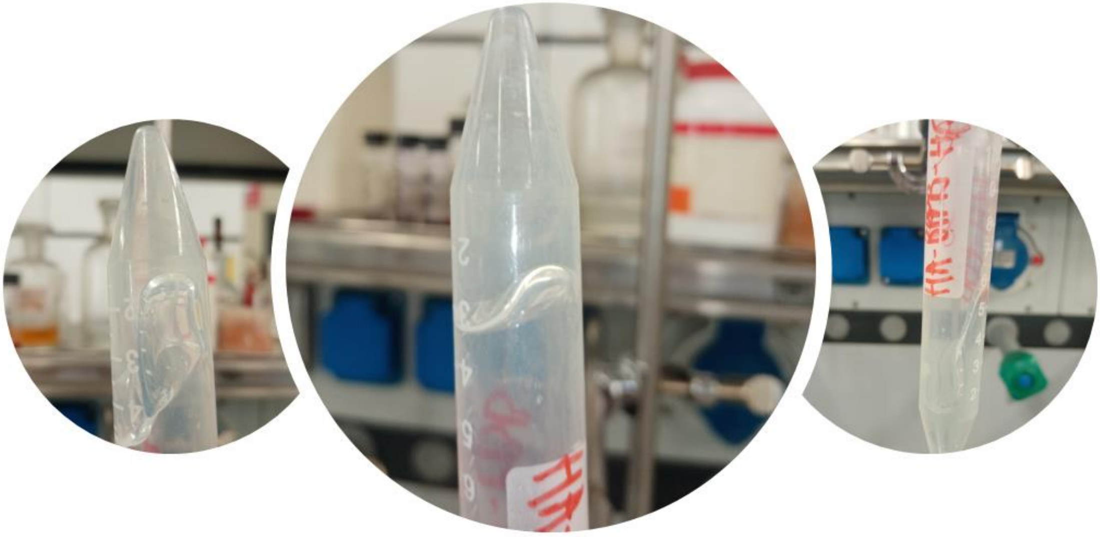

Figure S13. Appearance of the soaked HA-BPBB-HG (gummy state).

Video S1: VID-20240824-WA0011.mp4

Video S2: VIDEO-2024-08-29-15-28-56.mp4

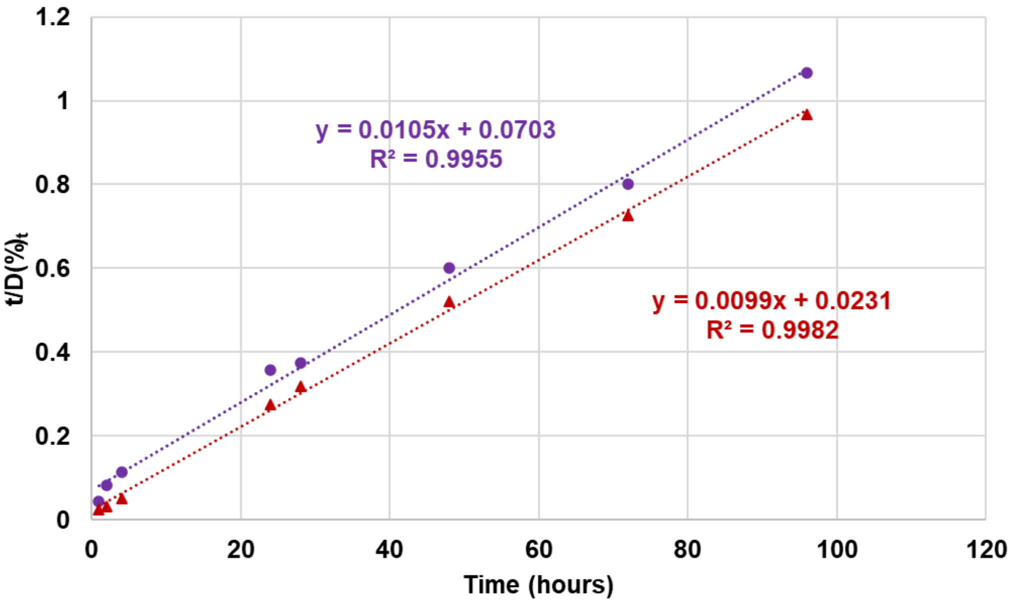

Figure S14. PSO kinetic model on biodegradation experiments.

Disclaimer/Publisher's Note: The statements, opinions and data contained in all publications are solely those of the individual author(s) and contributor(s) and not of MDPI and/or the editor(s). MDPI and/or the editor(s) disclaim responsibility for any injury to people or property resulting from any ideas, methods, instructions or products referred to in the content.
